# Calcium Carbonate from *Anadara granosa* Shells Stimulates FGF2, TGF-β1, and Collagen Type 1 Expression in Rat Dental Pulp

**DOI:** 10.1055/s-0044-1793842

**Published:** 2024-12-30

**Authors:** Widya Saraswati, Azlin Noor Yahya, Yovita Yonas, Ganiezha Cindananti, Nabiela Rahardia, Rania Rizka Ramadani, Venny Lusanda Ambarwati, Devy Putri Kusumawardhani, I Gede Marantika Yogananda Sutela, Larasati Kianti Putri, Brian Dwi Baskoro, Putu Krisnanda Pratama, Dawailatur Rahman Setiady

**Affiliations:** 1 Department of Conservative Dentistry, Faculty of Dental Medicine, Universitas Airlangga, Surabaya, Indonesia; 2 Department of Restorative Dentistry, Faculty of Dentistry, Universiti Malaya, Malaysia; 3 Postgraduate Program of Conservative Dentistry Specialist, Faculty of Dental Medicine, Universitas Airlangga, Surabaya, Indonesia; 4 Undergraduate Program, Faculty of Dental Medicine, Universitas Airlangga, Surabaya, Indonesia

**Keywords:** calcium carbonate, dentine, growth factor, FGF2, TGF-β1, collagen type 1

## Abstract

**Objectives** Calcium carbonate (CaCO _3_ ), a major inorganic component in bones and teeth, offers potential protection against demineralization. This study investigates the effect of CaCO _3_ from *Anadara granosa* shells on the expression of fibroblast growth factor 2 (FGF2), transforming growth factor-β1 (TGF-β1), and collagen type 1 in the rat dental pulp.

**Materials and Methods** The first maxillary molars of *Rattus norvegicus* were perforated and subsequently pulp capped with CaCO _3_ extracted from *A. granosa* shells. The cavities were then filled with glass ionomer cement, while the control group received calcium hydroxide (Ca(OH) _2_ ). Teeth were extracted after 7 and 14 days of treatment, and the expression of FGF2, TGF-β1, and collagen type 1 in the dental pulp was analyzed using immunohistochemistry staining.

**Results** The group treated with CaCO _3_ from *A. granosa* shells exhibited significantly higher expression of FGF2, TGF-β1, and collagen type 1 in the dental pulp at both 7 and 14 days compared with the group treated with Ca(OH) _2_ ( *p* < 0.01).

**Conclusion** The application of CaCO _3_ derived from *A. granosa* shells enhances the proliferative phase in the dental pulp after pulp perforation and perhaps promotes reparative dentine formation.

## Introduction

Dental caries is an avoidable, persistent, biofilm-mediated condition influenced by various dietary factors. 1 The primary cause of dental caries is an imbalance in oral biofilm, which occurs when fermentable dietary carbohydrates linger on tooth surfaces for an extended period. The impact of dental caries on the dentine is intricately linked to bacterial by-products like metabolites, toxins, and cell wall elements resulting in the dissolution of organic and inorganic components. 2 The destruction of dentin during the caries process results in exposure of the dental pulp, leading to a main symptom like pain. 3 To prevent dental pulp destruction and maintain vitality, vital pulp therapy was performed. 4 The primary goals of vital pulp therapy are to preserve the pulp vitality and induce the formation of a protective tissue called reparative dentine. 5 This treatment comprises various techniques, such as pulp capping, which involve applying a biocompatible material to the remaining innermost layer of affected dentine to prevent exposure of dental pulp and induce the reparative dentine. 6

The dental pulp is mainly composed of fibroblasts. 7 When the dental pulp is exposed, some fibroblast and odontoblast cells become damaged. To recover, the process of stimulating cell migration, proliferation, and tissue matrix formation is required. 8 During this process, several growth factors are needed to recover dental pulp, like transforming growth factor-β1 (TGF-β1) and fibroblast growth factor 2 (FGF2). 9 FGF2 is a potent mitogenic factor for fibroblasts. FGF2 expressed in odontoblast and osteoblast cells regulates the mineralization process through the proliferation, homing, and migration of dental pulp cells. 10 In the dental pulp, collagen matrix synthesis is induced by TGF-β1. Type I collagen, a significant component of dentine (around 90%), acts as a scaffold for mineralization. Type I collagen in odontoblast cells is considered an early marker of the reparative dentine formation process. 11

Calcium hydroxide (Ca(OH) _2_ ) is commonly used as a pulp capping agent due to its antibacterial properties. 12 However, it has several drawbacks, including pulp cell apoptosis, poor marginal adaptation to dentine, limited long-term seal against microleakage, potential irritation, pulpal calcification, and obliteration of the root canal. These limitations have led to the exploration of alternative treatment options, such as natural or biogenic materials, including calcium carbonate (CaCO _3_ ). *Anadara granosa* , widely consumed in Indonesia, produces a substantial amount of shell waste. The shells primarily consist of CaCO _3_ , constituting approximately 98 to 99% of the material. CaCO _3_ , a major inorganic component in bones and teeth, offers potential protection against demineralization. The application of CaCO _3_ from the *A. granosa* shell in the dentin–pulp complex affects inflammatory response by decreasing nuclear factor kappa B (NF-κB) expression 13 and increasing vascular endothelial growth factor A (VEGF-A) expression. 14 For this reason, utilizing CaCO _3_ from *A. granosa* shells holds promise as a potential material for inducing and expediting the process of dentin reparative. This study was performed to analyze the effect of CaCO _3_ from *A. granosa* shells on dental pulp regeneration, especially in the proliferative phase, by analyzing the expression of FGF2, TGF-β1, and collagen type I, which are major components in the formation of reparative dentine.

## Material and Methods

### CaCO _3_ from *Anadara granosa* Shell

The production and formulation of CaCO _3_ from *A. granosa* shell in this study followed the previous study by Saraswati et al. The CaCO _3_ paste was obtained by mixing the CaCO _3_ powder with sterile water at a ratio of 3:1. 15

### Animal

Four groups of healthy male *Rattus norvegicus* rats, with a weight of 300 to 350 g, were used as animal models. Each group consisted of 10 *R. norvegicus* rats. The protocol of this study design was approved by the Health Experiment Committee, Faculty of Dental Medicine, Universitas Airlangga, Indonesia, with registration number 558/HRECC.FODM/XII/2020. 465/HRECC.FODM/VII/2021, and 782/HRECC.FODM/X/2022.

### Tooth Cavity

Class I tooth cavity was created in the first maxillary molar using a diamond burr (diameter of 0.84 mm) with low-speed turbine with copious irrigation of sterile normal saline. The cavity depth was measured as 1 mm. The pulp was then exposed with a sterile sharp explorer. 16 Before the procedure, the animals were given 0.2 mL/kg body weight combined with anesthesia of ketamine hydrochloride and diazepam with a weight ratio of 10:1.

Following the perforation, the cavity was rinsed with a sterile saline solution and dried with cotton pellets. The CaCO _3_ powder was weighed using an analytical balance. The preparation was made with a 3:1 ratio by mixing 0.03 g of nano CaCO _3_ and 0.01 mL of distilled water. The powder and liquid mixture was stirred using a cement spatula until homogeneous. CaCO _3_ from the *A. granosa* shell was administered using a microbrush as pulp capping, then the cavity was filled with glass ionomer cement (Cention N, Ivoclar Vivadent, Schaan, Liechtenstein). In the control group, the tooth cavity was administered Ca(OH) _2_ and was filled with glass ionomer cement. The teeth in each group were extracted after 7 and 14 days accordingly.

### FGF2, TGF-β1, and Collagen Type 1 Expression

Histological assessment was done by doing immunohistochemistry staining for FGF2, TGF-β1, and collagen type 1 expression by fibroblast cells, which was quantified using a manual counting method under a microscope with ×1,000 magnification in 20 fields of view. Each field contained around 1,500 cells. The slides were coded, and their codes were covered and replaced with random new numbers to ensure that the examiner was blinded to the sample groups. After completing the counts, the examiner averaged the results. The primary antibodies were FGF2 (mouse monoclonal, Santa Cruz Biotechnology), TGF-β1 (mouse monoclonal, Santa Cruz Biotechnology), and collagen type 1 (mouse monoclonal, Santa Cruz Biotechnology). The secondary antibody was used in the 3,3′-Diaminobenzidine (DAB) system (Universal HRP Excell Stain, Biogear, Life Science). The counterstain used was hematoxylin 560 (Leica Biosystem).

### Statistical Analysis

The data on the expression of FGF2, TGF-β1, and type 1 collagen were subjected to analysis for distribution (Shapiro–Wilk test) and homogeneity (Levene's test). Additionally, an independent *t* -test was conducted to identify differences within each group, with a significance level of *p* ≤ 0.05.

## Result

### FGF2 Expression

**Fig. 1:**
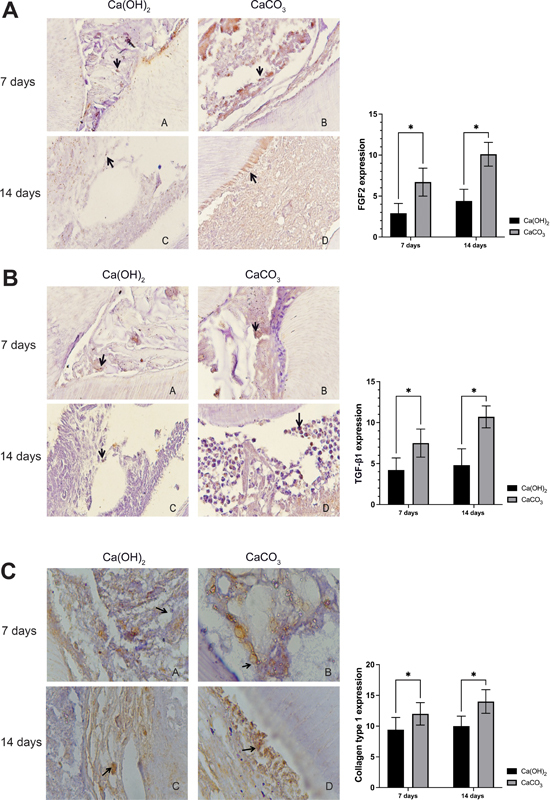
The immunohistochemistry staining of dental pulp tissue after being treated with CaCO _3_ from the *Anadara granosa or* Ca(OH) _2_ . (A) FGF2 expression (B) TGFβ1 expression (C) Type-1 collagen expression. Asteric indicated a significant difference compared indicated group with an independent t-test. * *p* < 0.05.

The FGF2 expression in the dental pulp treated by CaCO _3_ from the *A. granosa* shell showed a higher expression than those treated by Ca(OH) _2_ after 7 and 14 days ( *p* < 0.05; Fig. 1A ). These findings suggest that the FGF2 expression increases significantly after 7 and 14 days post CaCO _3_ application and further promotes the proliferation and migration of new cells to the injury site and supports the differentiation of mesenchymal stem cells, which, in turn, enhances the development of vascular tissues, fibroblasts, and osteoblasts.

### TGF-β1 Expression

The TGF-β1 expression in the dental pulp treated by CaCO _3_ from the *A. granosa* shell showed a higher expression than those treated by Ca(OH) _2_ after 7 and 14 days ( *p* < 0.05; Fig. 1B ). These findings suggest that the TGF-β1 expression increases significantly after 7 and 14 days post CaCO _3_ application and initiates the reparative process in pulp tissue by inducing the expression of growth factors

### Type 1 Collagen Expression

Similar to FGF2 and TGF-β1 expressions, the type 1 collagen expression was higher in the group treated by CaCO _3_ from the *A. granosa* shell than those treated by Ca(OH) _2_ after 7 and 14 days ( *p* < 0.05; Fig. 1C ). These findings suggest that type 1 collagen expression increases significantly after 7 and 14 days of CaCO _3_ application and acts as a bone matrix, which facilitates the process of dentinogenesis.

## Discussion

CaCO _3_ from the *A. granosa* shell in the dentin–pulp complex has shown an anti-inflammatory effect by decreasing the NF-κB expression 13 and interleukin-10 (IL-10) expression 17 and increasing vascularization by increasing the VEGF expression. 14 As a pulp capping material, CaCO _3_ from the *A. granosa* shell has shown its anti-bacterial effect, 18 and can used as bone scaffold materials. 19 The main effect of CaCO _3_ from the *A. granosa* shell is provided by hydroxyapatite. The hydroxyapatite content in CaCO _3_ from the *A. granosa* shell approximates 60% similarity to the chemical structure of the inorganic material in dentine. Hydroxyapatite, the principal form of calcium within the human body, constitutes the inorganic structure of dentine, comprising Ca ^2+^ and PO _4_^2−^ . Hydroxyapatite in conjunction with collagen bestows structural integrity to the dentine tissue matrix. 13 CO _3_^2−^ can substitute for the groups composing hydroxyapatite. The interaction of CaCO _3_ , which yields Ca ^2+^ and CO _3_^2−^ ions, with the PO _4_^3−^ present in the residual dentine and vital pulp can form carbonate apatite (CO _3_ Ap). Ca ^2+^ functions as a messenger mediating cellular responses to a variety of stimuli, such as proliferation, motility, secretion, and neurotransmission, 13 while CO _3_ Ap plays a role in the mineralization process of dentine. It is anticipated that the formation of carbonate apatite could enhance the mineralization of dentine and the creation of a hard tissue barrier, culminating in the development of reparative dentine. 20

Since the study of the effect of antibacterial and anti-inflammation in the dental pulp of CaCO _3_ from the *A. granosa* shell has been reported, the present research has shown its effect on several growth factors that support the proliferation phase. The first growth factor was TGF-β1, which can be attributed to the anti-inflammatory properties of CaCO _3_ . Ca ^2+^ from CaCO _3_ can inhibit the inflammatory processes in dental pulp via activation of the ERK1/2 pathway through calcium sensing receptors (CaSRs). This inhibition later inhibits the phosphorylation of the IκB protein and the activation of NF-κB. NF-κB inhibition results in decreased activation and production of proinflammatory cytokines, especially in M1, consequently decreasing inflammation. 21 On the other hand, to maintain the homeostasis, the M2 phenotype was activated and produced several growth factors and anti-inflammatory cytokines like TGF-β1. A significant upregulation of TGF-β1 expression was observed in the treatment with CaCO _3_ . This suggests that the expression of TGF-β1 naturally escalates in the reparative process postinjury and that CaCO _3_ administration hastens this reparative process. TGF-β1 is a multifunctional cytokine that plays a vital role in the reparative process following injury, being integral in inflammation, progenitor cell recruitment, cell proliferation, and differentiation. 22

The activation of the proliferative phase as a homeostatic tissue response involves the synthesis of anti-inflammatory cytokines, notably TGF-β1, which initiates the reparative process in pulp tissue. During tissue repair, TGF-β1 interacts with TGF-β receptors, leading to the phosphorylation of Smad2/3. This activation induces the expression of growth factors such as FGF2, which promotes the proliferation and migration of new cells to the injury site. Pulp cells, including progenitor cells and fibroblasts, differentiate into odontoblast-like cells, replacing damaged odontoblasts. 23 By increasing the levels of TGF-β1, the proliferation phase is characterized by enhanced proliferation and survival of resident cells in the dental pulp, 24 particularly fibroblasts and collagen-producing cells. 25

As a result of the increased expression of TGF-β1, fibroblasts in the dental pulp produce FGF2, which further promotes cellular proliferation. 26 This condition was also observed in this study, where the application of CaCO _3_ from *A. granosa* shells increased the expression of FGF2. Additionally, the application of CaCO _3_ from *A. granosa* shells was associated with increased expression of collagen type I.

As an anti-inflammatory agent, CaCO _3_ stimulates the release of FGF2 to reduce inflammation and support the differentiation of mesenchymal stem cells. This, in turn, enhances the development of vascular tissues, fibroblasts, and osteoblasts. Vascular tissues facilitate the migration of mesenchymal stem cells to defect areas, providing essential oxygen and nutrients. Fibroblasts and osteoblasts promote collagen formation, serving as a bone matrix in the process of osteogenesis. 27

In line with the FGF2 expression, the collagen type 1 expression notably increased after treatment with CaCO _3_ from *A. granosa* shells. The higher collagen type 1 expression will lead to a higher deposition of collagen in dental pulp, indicating the occurrence of dentinogenesis. During this phase, the remodeling of type III collagen synthesis transitioned to the ribbon-shaped type I collagen and was characterized by enhanced tensile strength. The reorganization and cross-linking of collagen arrangements during the remodeling phase contributed to increased tissue strength and density, leading to a denser fibril arrangement. 28 Furthermore, collagen fibers often underwent mineralization, leading to the start of the reinforcement processes. During this process, collagen fibers were poised to enter the maturation phase, where the collagen level stabilized between deposited and degraded collagen. 29 The increased expression of collagen type 1 following treatment with CaCO _3_ from *A. granosa* shells indicated that materials could stimulate the formation of reparative dentine, with CaCO _3_ exhibiting a superior capacity to induce collagen type 1 expression. Thus, CaCO _3_ derived from *A. granosa* shells is anticipated to serve as an alternative pulp capping material to initiate the deposition of dentine tissue, facilitating healing through the formation of reparative dentine.

## Conclusion

The application of CaCO _3_ derived from *A. granosa* shells enhances the proliferative phase in the dental pulp after pulp perforation. These findings provide compelling evidence of the efficacy of CaCO _3_ derived from *A. granosa* shells in promoting the formation of reparative dentine.
